# Depression Is Associated with an Increased Risk of Subsequent Cancer Diagnosis: A Retrospective Cohort Study with 235,404 Patients

**DOI:** 10.3390/brainsci13020302

**Published:** 2023-02-10

**Authors:** Hannah Mössinger, Karel Kostev

**Affiliations:** 1 Epidemiology, IQVIA, 60549 Frankfurt, Germany; 2 Department of Gynecology and Obstetrics, Philipps-University, 35037 Marburg, Germany

**Keywords:** cancer, depression, outpatients, general physicians

## Abstract

**Highlights:**

**What are the main findings?**

- Our findings indicate patients with depression have an increased risk of cancer, ranging from 10% to 39% increased risk depending on the type of cancer.
- Cancer risk was highest in patients with depression for lung, GI, breast, and urinary cancer.

**What are the implications of the main findings?**

- Our findings elucidate the rarely investigated direct impact of depression on cancer risk.

**Abstract:**

**Background:** Depression and cancer share common risk factors and mechanisms of disease. The current literature has not explored the effect of depression on cancer risk. We assessed the difference in cancer risk in patients with and without depression in a large cohort in Germany. **Methods:** We compared cancer risk and incidence in patients with and without depression aged 18 or above diagnosed between 2015 and 2018 documented in the Disease Analyzer Database. Patients from a comparator group were matched 1:1 to patients with depression based on propensity scores. Patients with previous bipolar disorder (F31), mania (F30) or schizophrenia (F20–29) and cancer diagnosis 3 years prior to index date were excluded. Analyses were stratified by cancer type, age group, and gender. **Results:** A total of 117,702 patients with depression were included and matched 1:1, resulting in a cohort overall of 235,404. 4.9% of patients with depression compared to 4.1% without depression received at least one cancer diagnosis over 3.9 years median follow-up. The depression group showed an 18% increase in risk for a cancer diagnosis overall, with largest increased risk in lung cancer (HR: 1.39 [1.21–1.60], *p* < 0.0001), cancers of the gastro-intestinal-tract (HR: 1.30 [1.15–1.46], *p* < 0.0001), breast (HR: 1.23 [1.12–1.35], *p* < 0.0001) and urinary (HR: 1.23 [1.06–1.43], *p* < 0.01). Similarly, the incidence of cancer diagnosis overall increased by 22% for depressed patients. IRs showed no difference across cancer types. **Conclusions***:* Depression increased the risk for cancer diagnosis consistently independent of the comparison method used. The potential mediating factors or shared mechanisms of the disease require further investigation.

## 1. Introduction

Unipolar depression causes the greatest burden on mental health globally as measured in disability-adjusted life years [1], and is the second most leading mental health disorder globally with a 12-month prevalence of 7–8%, with women showing approximately double the risk compared to men [2]. Depression is highly undertreated worldwide [3] and linked to numerous chronic somatic comorbidities [4] including cancer. Depression is present in 3–31% of patients with cancer, with prevalence varying by type of cancer, sex of the patient, and other factors [5,6,7]. Furthermore, depression has a negative impact on most somatic conditions [8,9], with cancer patients with comorbid depression showing reduced treatment compliance, increased overall mortality, and shorter survival due to reduced adherence [10,11]. Depression has been linked to cancer via multiple pathways of association: via behavior associated with depression and cancer, as well as shared risk factors and disease mechanisms.

Habits such as smoking and substance abuse, as well as conditions such as obesity, are known to be harmful to general health and are frequent in patients with psychiatric illness [12,13]. Substance abuse with alcohol, tobacco, or illicit substances can cause physical illnesses such as cancer [14], linked via mutagenic effects or dysregulation of the immune system [15,16]. 

Previous studies have focused on the association between cancer and depression diagnosis and their joint impact on all-cause mortality [17,18,19]. However, the impact of depression on cancer diagnosis is less investigated. Most studies examined whether a previous depression diagnosis was linked to cancer in women with breast cancer [11,20,21], rather than the incidence of cancer of any type in patients with depression. Onitilo et al. (2006) examined the impact of depression across a variety of cancer sites with adequate control cohorts to differentiate the effect of depression on cancer and all-cause mortality in patients in the USA [22]. Wang et al. (2020) conducted a meta-analysis of cohort studies and found within 21 studies with more than 1,6 million patients an increased risk for cancer incidence in patients with depression [23], however with large heterogeneity among the studies examined impacting the interpretability of the results.

The goal of the present study is to evaluate the association between depression and the subsequent cancer diagnosis in a large cohort in Germany based on a 10-year follow-up period. 

## 2. Method

### 2.1. Database

The data used for this retrospective cohort study was taken from the Disease Analyzer (DA) database (IQVIA), a national database of anonymized diagnosis and prescription information obtained from general and specialist practitioners in Germany [24]. The sample of physicians represents around 3% of practices in Germany. The sampling method for the Disease Analyzer database is based on summary statistics from all doctors in Germany published yearly by the German Medical Association. IQVIA uses these statistics to determine the panel design according to the following strata: specialist group, German federal state, community size category, and age of physician. Data quality and accuracy is monitored continuously based on numerous criteria (documentation completeness, linkage between diagnosis and prescription). The DA database contains anonymized information on patient demographics (age, gender, diagnosis type) as well as disease information including ICD-10 diagnosis code, diagnosis, and visit dates. The data are obtained directly from the electronic records of participating sites and physicians, which are then encrypted to protect data privacy before monthly transmission to IQVIA (Frankfurt, Germany). The representativeness of the sample population in this database is assessed annually by IQVIA and is found to be representative of primary care in Germany [24]. 

Due to the anonymized nature of the data included in DA, in compliance with German laws on data protection, no ethics approval is needed for analyses based on secondary data. This database has been used in previous studies on depression and cancer [17,25]. 

### 2.2. Study Outcomes and Variables

The index date was determined per person as the date of first depression diagnosis or physician visit date as applicable (see Section 2.3. Study Population). Baseline observation time and follow-up time were calculated as the difference (in years) from index date until first or last visit date, respectively, documented for each patient in the DA database. Visit frequency was calculated by determining the number of quarters in which each patient had a documented visit to their physician on average per year of observation time, resulting in values from 0–4. 

### 2.3. Study Population

**Figure 1:**
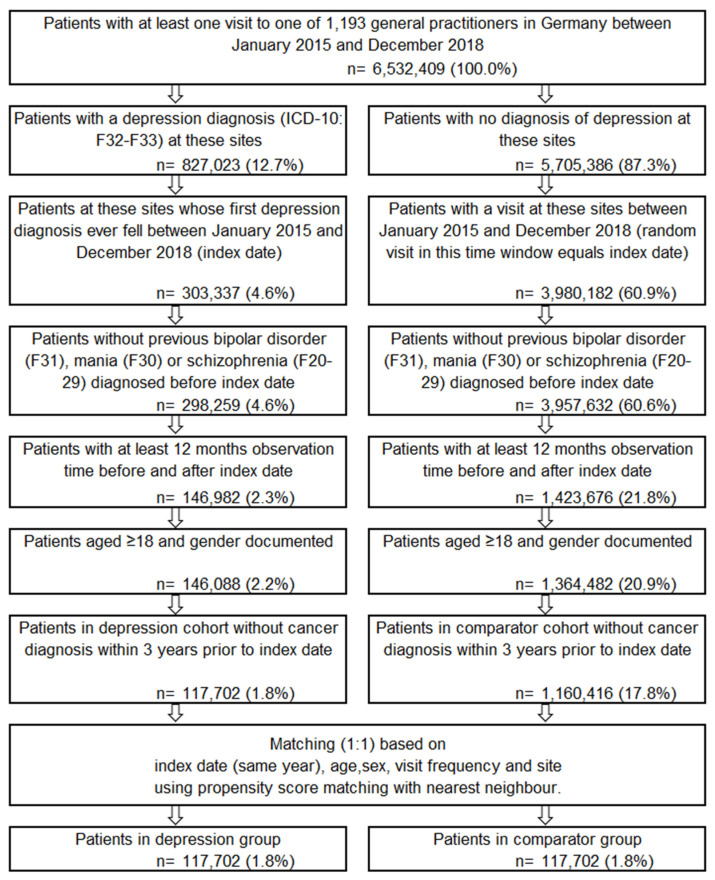
Attrition diagram of study cohort.

Patients were included in the cohort from the DA database if they had a physician visit within the inclusion window of January 2015 to December 2018, allowing for a follow-up period of at least 3 years per patient at time of data extraction in November 2021. Patients with depression were determined as patients with a first depression diagnosis (ICD-10 codes F32, F33) in this inclusion window, while the patients in the comparator group (CG) were chosen based on at least one visit within this time and no depression diagnosis at any point, respectively. The index date was determined as the first depression diagnosis in the 303,337 patients in the depression group (4.6% of patients included in the database), while the index date of the 3,980,182 patients (60.6% of patients included in the database) in the comparator group was a randomly selected visit which fell within the inclusion window. In both groups, any patients with a diagnosis of bipolar disorder (F31) or schizophrenia (F20–F29) before index date were excluded. Only patients with a minimum 1-year baseline and follow-up observation period, aged ≥ 18 years, and with gender documented were further selected. The resulting 117,702 patients with depression (1.8% included from the database) were matched 1:1 to patients from the comparator group using propensity score matching of nearest neighbors with a caliper of 0.2. The propensity score was based on index date (same year as patient in depression group), age, gender, visit frequency, and site. Visit frequency was included in the matching to remove any bias of chronically ill patients having more frequent physician visits and to equalize the detection rate of cancer between groups. This resulted in 117,702 patients with depression (1.8% included from the database) matched with 117, 702 patients in the comparator group (see Figure 1). Matching was based on propensity scores calculated from diagnosis year, age, gender, visit frequency (quarters per year with one visit), and site, with matched patients determined using 1:1 matching of nearest neighbors with a caliper of 0.2.

### 2.4. Statistical Methods

All analyses were conducted using SAS (SAS Institute) Version 9.4. Descriptive analyses were conducted by reporting the proportion of patients for categorical variables, mean, and SD for continuous variables. Time until cancer diagnosis and risk of cancer diagnosis was assessed using Kaplan–Meier curves and median time to event, as well as by calculating the hazard ratio (HR) between patients with and without depression. Analyses were stratified by age (18 to 30, 30–90 years in 10-year increments), sex (male and female), type of cancer diagnosis (skin, breast, gastro-intestinal, prostate, lymphatic or blood, lung, urinary, see Table S1 for ICD-10 codes used per cancer diagnosis group). To assess whether potential undetected cancers in both cohorts may have influenced the diagnosis of depression, we conducted a sensitivity analysis where any cancer diagnosis within the first twelve months of the index date was disregarded.

The incidence rate (IR) of cancer was calculated using the total number of events per cancer diagnosis (overall and by type) in proportion to person-years of exposure time. Person time was calculated as the time (in years) from index date until date of last visit documented. The 95% CI was calculated by fitting a Poisson model in SAS using general linear modeling of least square means. The difference in incidence rate between groups was calculated using the rate ratio of the least square means with general linear models. Analyses were stratified by age group and sex. Where numbers allowed, the incidence rate per type of cancer diagnosis was calculated.

## 3. Results

### 3.1. Baseline Characteristics

The demographic and clinical characteristics of matched groups of patients with and without depression are shown in Table 1. Patients with major depression matched with patients without depression showed no significant difference in sex, age distribution, physician visit frequency, most common somatic comorbidities. Groups showed differences in pre-index and follow-up observation time as well as distribution of insurance type and diagnosis year and comorbidities. Patients with depression had a moderate diagnosis in 23.1% of cases, 10.4% were mild and 5.6% were severe. The majority of 60.9% fell into “other or undefined” severity. Patients with MD showed more frequent psychiatric comorbidities (10.4% MD, 2.6% CG) including anxiety disorder (F40–43, 2.2% MD, 0.8% CG) and neuroses and somatoform disorders (F45–48, 1.7% MD, 0.7% CG). 

### 3.2. Cancer Diagnosis Risk

The proportion of patients with a cancer diagnosis recorded in both groups is shown in Table 2. A total of 5781 patients with depression received at least one cancer diagnosis (4.9%) compared to 4822 patients in CG (4.1%). The number of events of cancer diagnoses increased from 0.5% in MD and 0.4% in CG in the age group 18–30 years to 12.6% in MD and 10.7% in CG in patients aged >80–90 years. Fewer events were observed in patients >90 years, with 11.6% in MD and 9.3% in CG. Women and men showed the same number of events in MD (4.9%), while there was a difference in sexes in CG (women 3.8% and men 4.5%). Skin cancer was the most common type of cancer diagnosis, with 1203 events (1% of patients) in MD and 1072 events (0.9%) in CG, followed by breast cancer (0.8% MD, 0.6% CG), gastro-intestinal (GI) cancers (0.6% MD, 0.4% CG), prostate cancer (0.4% in MD and CG), cancers of the blood and lymph (0.4% MD, 0.3% CG), lung cancer (0.4% MD, 0.3% CG) and urinary cancers (0.3% MD and CG). Not enough events were recorded to reach the median in any group, therefore median and 95% CI are not reported here.

**Figure 2:**
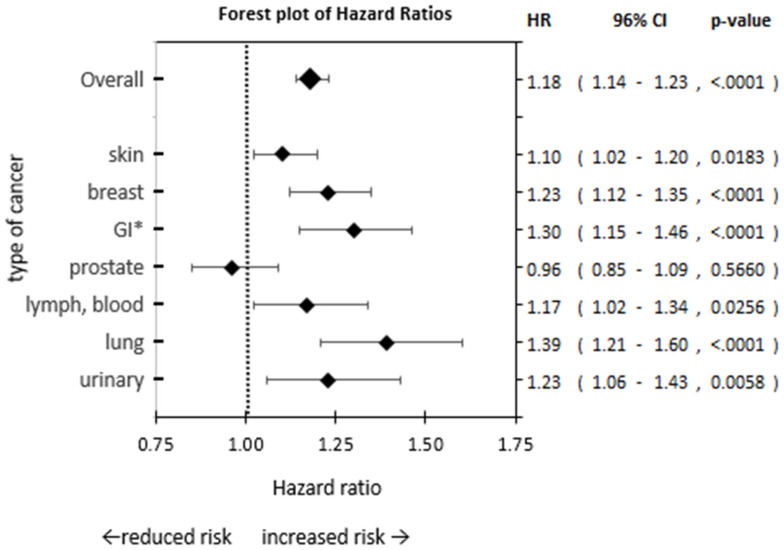
Forest plot of hazard ratios. Risk of cancer diagnosis was compared between patients with depression and patients without depression (ref) across subgroups by cancer type. GI*: gastro-intestinal.

To compare the risk between groups, hazard ratios were calculated overall and per subgroup. Patients with depression showed an increased risk for any cancer diagnosis compared to patients without depression (HR: 1.18 [1.14–1.23], *p* < 0.0001), which was consistent across age groups, but only reached significance from age groups >40–50 years (HR: 1.49 [1.26–1.76], *p* < 0.0001) until >80–90 years (HR: 1.18 [1.10–1.28], *p* < 0.0001). Men showed less of a difference in risk of cancer diagnosis due to depression (HR: 1.08 [1.02–1.15], *p* = 0.0112) than women (HR: 1.27 [1.21–1.33], *p* < 0.0001). The types of cancers showing the greatest difference in risk were cancers of the lung (HR: 1.39 [1.21–1.60], *p* < 0.0001), GI (HR: 1.30 [1.15–1.46], *p* < 0.0001), breast (HR: 1.23 [1.12–1.35], *p* < 0.0001), and urinary (HR: 1.23 [1.06–1.43], *p* < 0.01). Cancers of the skin (HR: 1.10 [1.02–1.20], *p* < 0.05) and lymph or blood (HR: 1.17 [1.02–1.34], *p* < 0.05) indicated some increased risk for patients with depression. Cancers of the prostate (HR: 0.96 [0.85–1.09], *p* = 0.057) showed no difference in risk between MD and CG (see Table 2, Figure 2).

Data from the sensitivity analysis excluding all events of cancer diagnosis within 12 months after the index date showed a lower risk for cancer diagnosis overall, by age group, gender, and type of cancer, but with similar trends as described above (Table S2, Figure S1).

### 3.3. Incidence of Cancer Diagnosis

To account for potentially multiple events of cancer diagnosis in this sample, the comparison of events and risk was also conducted using incidence rates. Table 3 shows the incidence rate within each subgroup of interest. The IR over 1000 person-years per group for any cancer diagnosis was 15.23 in patients with depression compared to 12.62 events per 1000 person-years in patients without depression. The rate ratio indicated a significant difference (1.22 [1.18–1.27], *p* < 0.0001). The incidence rate ranged from 1.58 in MD and 1.17 in CG in ages 18–30 years to 41.23 in MD and 32.53 in CG in patients >90 years, albeit with significant rate ratios only seen in age groups of over 40 to 50 years, over 50 to 60 years, over 60 to 70 years and over 80 to 90 years. Similar to the HRs described above, the magnitude of the significant rate ratios decreased from 1.52 in the over 40- to 50-year-olds to 1.18 in the over 80- to 90-year-olds (*p* < 0.0001 for all). The incidence of cancer risk did not differ between men and women in the MD group (15.45, 15.10, respectively), while in the CG men showed a higher incidence of cancer (14.30) compared to women (11.50). The rate ratio indicated a larger effect of depression on cancer risk in women (1.32 [1.26–1.39]) compared to men (1.09 [1.03–1.16]). Within the group of patients with depression, the incidence of cancer diagnosis risk lay between 14.24 and 16.77 per 1000 person-years depending on the severity of the depression diagnosis. The incidence of cancer diagnosis by cancer type differed significantly between MD and CG for skin cancer (2.62 vs. 2.37, *p* < 0.05), but IRs showed little difference despite a significant test statistic for breast, GI, lymph and blood, lung, and urinary cancer (see Table 3).

## 4. Discussion

In this retrospective study based on data from 235,404 patients treated in general practices in Germany between 2015 and 2018, matched 1:1 between patients with depression and patients without depression, a subsequent cancer diagnosis occurred in 4.9% of patients with depression compared to 4.1% without, indicating an 18% increase in the risk of a subsequent cancer diagnosis in depressed patients overall. The increase in risk was consistent across age groups examined, ranging from an 18–49% increase in risk depending on the age group for patients with depression. Depression was also associated with a higher rate of cancer risk in specific cancer types examined, specifically in the skin, lymph, blood, urinary, breast, and lung. Based on the latest reports on cancer incidence in Germany, 2–8% of patients were expected to receive a cancer diagnosis, with at least 4% of cases expected in the over 50-year-olds [26], which corresponds to our results shown here. 

Lung cancer, and cancers of the GI, breast, and urinary system showed the greatest difference in risk by HRs between groups with and without depression. Incidence rates and rate ratios did not reflect the trends in relative risk assessed by HR. However, rate ratios of lung, breast, and GI cancers remained statistically significant, indicating a difference in incidence rates between groups with and without depression and indicating a need to further investigate mediating factors. However, the DA database cannot shed light on the impact of potential mediating factors between depression and cancer.

Results from the increased risk measured by HRs are in line with a meta-analysis which indicated an increased risk of cancer diagnosis in depression and anxiety overall, and specifically for cancers of the lung and skin [23]. Wang et al. (2020) assessed the association between depression and anxiety across 21 cohort studies with more than 1,6 million patients. They found an increased risk for cancer incidence in patients with depression (RR 1.13, 1.06–1.19 95% CI), which was increased if only clinically diagnosed patients were assessed, or if a period of more than 10 years was available for follow-up assessment [23]. Interestingly, the effect on cancer mortality was higher (RR 1.21, 1.16–1.36 95% CI), indicating depression’s effect on mortality is mediated also by behavioral effects, while the incidence of cancer may be explained more directly by depression and anxiety itself. However, Wang et al. did not report the effect of depression on cancer risk alone, and the studies examined showed a high degree of heterogeneity, probably due to the inclusion of both clinically diagnosed and symptom-scale-based assessments of depression, which limits the comparability of their results to ours. 

Other previous studies investigating the impact of depression on cancer evaluated the combined effect of depression in cancer patients on mortality and focused primarily on the relationship between depression and breast cancer. Goodwin et al. (2004) investigated in over 24,000 patients across the USA how a depression diagnosis within 2 years before a breast cancer diagnosis impacted cancer-specific and all-cause mortality. The 7.5% of patients in their cohort with previous depression showed a 42% higher risk for cancer-specific and a 39% increased risk for all-cause mortality. They also identified that depression itself had an impact on 3-year mortality independent of whether patients received recommended or non-guideline treatment, indicating that depression had an impact beyond behaviorally mediated effects such as treatment compliance [20]. How depression severity mediates this effect could not be assessed as disease severity in this cohort was not reported. Hjerl et al. (2003) showed a similar increased risk of 21–42% of all-cause mortality in the 0.5–0.7% of breast cancer patients with a pre-operative depression diagnosis out of over 20,000 Danish patients [21], as did Gathinji et al. (2009) with 41% increased risk in mortality in 1052 patients with malignant brain astrocytoma who had pre-operative depression (4.6% percent of the study cohort) [27]. The increased risk of cancer mortality was highest at 50% in 343 of 11,065 patients with 5-year cancer survival within Korean national claims data and was further increased in subgroups of cancers related to obesity and cancers of the GI tract (59% and 58% increased risk, respectively) [11].

Investigating the impact of depression on the increased risk of certain types of cancers indicates potential key mediating factors, such as risk-increasing behaviors, or shared mechanisms of disease. The types of cancer showing the greatest increased risk due to depression (lung cancer, cancers of the GI, breast, and urinary system) are known to be strongly associated with risky behavior such as smoking [28,29,30,31,32], or conditions such as obesity [33], which are known to be strongly associated with depression [4,13,34,35]. Conversely, we saw no difference in risk between groups on prostate cancer, which is also associated with smoking [36].

Potential shared mechanisms of disease to explain the linkage between depression and cancer may involve an inflammation-induced activation of the chronic stress response and associated dysregulation in downstream effectors associated with depression and carcinogenesis, as well as shared maladaptive behaviors which further aggravate this potential shared mechanism of disease. 

Depression and cancer are both associated with increased inflammation, which activates the hypothalamic—pituitary—adrenal (HPA) axis, which mediates among other things the body’s metabolic and stress response pathways. Chronic illnesses associated with pain and inflammation are more common in patients with depression than in patients without depression [37], and chronic inflammation has been shown to predispose to certain types of cancer [38]. Downstream effectors of this pathway include pro-inflammatory entities such as cytokines which are upregulated and downregulated anti-cancer immune cells which thereby benefit the development of cancer [19,35,38]. Further downstream effects impact cortisol-binding receptors, leading to increased cortisol; hypercortisolism is implicated in appetite and fat deposition and is elevated in both depression and obesity [35]. Cytokines can further increase 5-HT and noradrenaline (NA) reuptake, reducing synaptic serotonin and NA which is linked directly to depressive behavior [19]. 

This immune stress pathway explains somewhat how maladaptive behaviors and risks such as obesity and drinking are shared across patients with depression and cancer, as these risks and behaviors tie into the immune stress pathway at various points. Understanding the impact inflammation, chronic stress, and their downstream regulators have on depression is yielding the first results for clinically relevant interventions. Within a phase 3 trial treating patients with depression with an anti-inflammatory agent, improved outcomes for depression patients on the Hamilton Scale for Depression (HAM-D) were not seen in the overall trial results comparing patients treated with anti-inflammatory agent infliximab compared to patients treated with placebo, but only in patients with high levels at baseline in inflammatory biomarker, i.e., baseline higher levels of chronic inflammation [39]. 

Other evidence linking depression and cancer does not directly implicate this pathway, or the mechanisms are not known, but still confirms the association between depression and cancer. Smoking is well-known to increase the risk of certain cancers, and studies show current smokers had an increased risk to have depression and developing depression compared to people who quit smoking or never smoked [13]. Sleep disorders are common in patients with depression [4] and show a bi-directional association with depression [34] as well as cancer [40], however, the mediating mechanisms in both diseases are currently not well understood. A contribution via the above-described stress–inflammation mechanisms is probable since sleep deprivation or tobacco can be seen as different forms of stress to the body, and both directly impact information and reward processing in the brain, which is altered in patients with depression.

It is known that chronic medical conditions lead to an increased risk of depression, but we have shown here that depression itself is associated with a higher risk of somatic diseases such as cancer. Depression has been shown to increase disease impacts of cancer due to reduced treatment adherence and worsening of disease symptoms, as well as increasing all-cause mortality and shortening cancer survival [10,11,41,42,43]. Survival and symptoms improved with reduced depression [44]. Understanding the key mediating risk factors allows for the identification of particularly high-risk depression patient groups or to identify target areas to support patient health and offset the risk of developing further comorbidities such as cancer.

### Limitations

The rates of observed cancer events over 4 years in this sample did not reflect expected rates reported by the German Centre for Cancer Registry Data, with 1% of patients in our sample affected by skin cancer compared to an annual expected incidence of 4.7 [26]. This difference may be accounted for by our sample selection. To allow for equal detection rates of both depression and comorbidities such as cancer, we used only data from GPs in the database. This discounts other specialties such as dermatologists, gynecologists, etc., which are expected to have a higher detection rate of cancers within their specialty. It also limits our observations in this dataset to cancers that have a longer survival time, as acute or severe cancer cases will most likely be attended by specialists or in a hospital setting. 

Our data does not allow a valid assessment of potentially mediating effects, including smoking or other substance abuse, on the observed difference in risk shown here by depression on different cancer types. However, the consistent results in the sensitivity analysis and across relative risk and rate ratio assessment indicate a robust trend towards an increased risk for specific cancer types. We did not adjust our HR model for whether patients received anti-depressive treatment. This, therefore, does not allow us to assess whether anti-depressive treatment and type of treatment had a mediating effect on the link between depression and cancer risk.

As most depression diagnoses had severity “other or undefined” (60.9%), this severely impacts the interpretability of these results and allows no valid interpretation of whether the incidence rate of cancer differed or remained the same depending on the severity of the depression. Similarly, no analysis of cancer severity and the difference in risk due to depression was possible, as most ICD-10 codes do not reflect cancer severity and this information was not collected in the database.

Due to the smaller number of events in patients under 40 years, potential age effects are masked by the high variability in the data, shown by the wide CI bands for HRs in Figure 2 The difference in risk decreased with age as the number of events increases, indicating less of an effect of depression on the risk of cancer. This may be due to the increase in comorbidities and decrease in general health with age, correlating with the risk of cancer [45], or due to an increase in depressive mood with age [46], reducing the difference between groups with and without depression. 

## 5. Conclusions

We have shown a link between depression and subsequent cancer diagnosis using matched cohorts from a retrospective database, the Disease Analyzer database, across a broad range of cancer indications. The increased risk of depression in various types of cancer showed both age and sex effects. The types of cancers most increased in risk as documented by GPs indicate a mediating effect of smoking, which cannot be further investigated in the database used here. The shared mechanisms of disease or shared risk factors in depression and cancer merit further investigation. 

## Figures and Tables

**Table 1:** Patient demographic and clinical characteristics by matched groups.

	Matched Groups				Group Difference (*p*-Values) ^+^
	Patients with Depression		Patients without Depression		
*N* Patients	117,702		117,702		-
Diagnosis year					
2015	26,740	22.7%	29,496	25.1%	-
2016	31,544	26.8%	27,825	23.6%	-
2017	29,848	25.4%	28,922	24.6%	-
2018	29,570	25.1%	31,459	26.7%	-
Sex					
Female	71,073	60.4%	71,138	60.4%	0.78 ^a^
Male	46,629	39.6%	46,564	39.6%	
Age					
Mean (SD)	55.0	18.4	55.0	19.9	0.8 ^b^
18–30	12,945	11.0%	17,096	14.5%	-
>30–40	16,133	13.7%	15,710	13.3%	-
>40–50	17,956	15.3%	15,186	12.9%	-
>50–60	26,018	22.1%	21,109	17.9%	-
>60–70	20,523	17.4%	19,188	16.3%	-
>70–80	10,826	9.2%	15,050	12.8%	-
>80–90	10,589	9.0%	11,603	9.9%	-
>90	2712	2.3%	2760	2.3%	-
Insurance type					
Statutory	112,369	95.5%	107,273	91.1%	<0.0001 ^a^
Private	5333	4.5%	10,429	8.9%	
Index diagnosis severity					
Mild	12,183	10.4%	0	0.0%	-
Moderate	27,165	23.1%	0	0.0%	-
Severe	6615	5.6%	0	0.0%	-
Other and undefined	71,739	60.9%	0	0.0%	-
None, missing	0	0.0%	117,702	100.0%	-
Visit frequency ^1^					
Mean (SD)	2.5	1.1	2.5	1.1	0.8 ^c^
Comorbidities ^2^					
Hypertension (I10)	1,440,870	15.4%	1,689,483	21.0%	-
Diabetes (E10–14)	462,626	4.9%	514,175	6.4%	-
Lipid metabolism disorder (E78)	304,267	3.2%	342,310	4.3%	-
Ischemic heart diseases (I20–I25)	236,564	2.5%	243,337	3.0%	-
Osteoarthritis (M15–M19)	164,548	1.8%	138,282	1.7%	-
Atrial fibrillation (I48)	96,077	1.0%	102,862	1.3%	-
Obesity (E65–E68)	51,108	0.5%	38,802	0.5%	-
Heart failure (I50)	79,010	0.8%	75,803	0.9%	-
Osteoporosis (M80–M81)	58,795	0.6%	56,608	0.7%	-
Dementia (F01, F03, G30)	34,190	0.4%	19,896	0.2%	-
Cerebrovascular disease (I60–63, G45)	29,052	0.3%	23,300	0.3%	-
COPD (j44)	123,727	1.3%	102,722	1.3%	-
Other, non-chronic	5,226,238	55.8%	4,411,909	54.9%	-
Other psychiatric disorders (F)	970,870	10.4%	207,620	2.6%	-
Neuroses, somatoform disorders (F45–48)	161,893	1.7%	57,996	0.7%	-
Anxiety disorders (F40–43)	206,737	2.2%	62,595	0.8%	-
Substance use (F10–F19)	76,484	0.8%	42,907	0.5%	-
Behavioral disorder with physical disfunction (F50–F59)	29,756	0.3%	17,419	0.2%	-
Other not specified (F99)	7981	0.1%	3126	0.0%	-
Baseline observation time (years) ^3^					
Mean (SD)	7.6	5.8	7.3	5.5	<0.0001 ^a^
Follow-up time (years) ^3^					
Mean (SD)	3.9	1.4	3.8	1.4	<0.0001 ^a^
Matching					
Mean caliper	0.8				-

^1^ Visit frequency given as quarters per year with at least one physician visit. ^2^ Comorbidities recorded any time before index date, multiple counts per patient possible. ^3^ Observation time calculated between first/last visit date documented and index date. ^+^ *p*-values were calculated for group differences based on chi-squared test (^a^), Kruskal–Wallis test (^b^) or t-test (^c^) for variables included in the matching algorithm (propensity score matching). COPD: Chronic obstructive pulmonary disorder.

**Table 2:** Hazard ratio of cancer diagnosis by group, subgroup, and type of cancer.

	Number of Events		Comparative Risk			
	Patients with Depression	Patients without Depression	Patients with Depression vs. Patients without Depression (Ref)			
Group	N Events (% ^1^)	N Events (% ^1^)	HR	96% CI (Lower–Upper)		*p*-Value
Any cancer diagnosis ^2^	4.9%	4.1%	1.18	1.14	1.23	<0.0001
By age group (in years)						
18–30	0.5%	0.4%	1.36	0.97	1.90	0.0725
>30–40	1.0%	0.7%	1.40	1.10	1.78	0.0062
>40–50	2.2%	1.4%	1.49	1.26	1.76	<0.0001
>50–60	3.8%	2.9%	1.30	1.17	1.43	<0.0001
>60–70	6.8%	5.3%	1.26	1.16	1.36	<0.0001
>70–80	10.4%	8.6%	1.22	1.13	1.32	<0.0001
>80–90	12.6%	10.7%	1.18	1.10	1.28	<0.0001
>90	11.6%	9.3%	1.26	1.07	1.48	0.0068
By sex						
Female	4.9%	3.8%	1.27	1.21	1.33	<0.0001
Male	4.9%	4.5%	1.08	1.02	1.15	0.0112

^1^: Percentage of events calculated as number of patients with at least one event divided by all patients within subgroup (by age, sex, type of cancer). ^2^: Any cancer diagnosis includes cancers of the breast, lung, prostate, skin, digestive system including stomach and colon, bladder and kidneys, female reproductive organs including uterus, cervix, ovaries, lymph and blood, and primary cancers of known location as well as malignant secondary of unknown primary origin.

**Table 3:** Incidence rate of cancer diagnosis by group, subgroup, and type of cancer.

	Incidence Rate Cancer Diagnosis ^1^		Rate Ratio ^2^			
	Patients with Depression	Patients without Depression	Patients with Depression vs. Patients without Depression (Ref)			
	IR/1000 Person-Years		Est.	95% CL (Lower-Upper)		*p*-Value
Any cancer diagnosis ^3^	15.23	12.62	1.22	1.18	1.27	<0.0001
By age group						
18–30	1.58	1.17	1.38	0.98	1.95	0.0639
>30–40	2.92	2.04	1.41	1.11	1.8	0.0057
>40–50	6.34	4.29	1.52	1.29	1.79	<0.0001
>50–60	11.03	8.43	1.35	1.22	1.49	<0.0001
>60–70	20.40	15.42	1.32	1.22	1.43	<0.0001
>70–80	31.65	25.39	1.24	1.15	1.33	<0.0001
>80–90	39.42	33.31	1.18	1.09	1.27	<0.0001
>90	41.23	32.53	1.27	1.08	1.49	0.0037
By sex						
Female	15.10	11.50	1.32	1.26	1.39	<0.0001
Male	15.45	14.30	1.09	1.03	1.16	0.0027
By depression severity						
Mild	14.99	-	-	-	-	-
Moderate	14.24	-	-	-	-	-
Severe	16.77	-	-	-	-	-
Other, undefined	15.51	-	-	-	-	-
By type of cancer						
Skin	2.62	2.37	1.12	1.03	1.22	0.0074
Breast	2.07	1.68	1.25	1.13	1.37	<0.0001
Gastro-intestinal	1.44	1.11	1.31	1.17	1.47	<0.0001
Prostate	1.05	1.09	0.98	0.86	1.11	0.7397
Lymph, blood	0.99	0.84	1.19	1.03	1.36	0.0144
Lung ^4^	1.03	0.74	1.23	1.21	1.24	<0.0001
Urinary	0.86	0.70	1.25	1.08	1.45	0.0031

Multiple events per patient possible. ^1^: Total number of events (cancer diagnosis) in proportion to person-years of exposure time in each group standardized to 1000 person-years. Person time was calculated as the time (in years) from index date until last visit date. The 95% CI was calculated by fitting a Poisson model in SAS using general linear modeling of least square means. ^2^: Difference in incidence rate between groups calculated using the estimated difference in log rates, i.e., the difference in least square means. ^3^: Any cancer diagnosis includes cancers of the breast, lung, prostate, skin, digestive system including stomach and colon, bladder and kidneys, female reproductive organs including uterus, cervix, ovaries, lymph, and blood, and primary cancers of known location as well as malignant secondary of unknown primary origin. ^4^: Rate ratio model was adjusted for comorbid COPD in 12 months pre-index date.

## Data Availability

Restrictions apply to the availability of this data. Data are available upon reasonable request.

## Supplementary Materials

The following supporting information can be downloaded at: https://www.mdpi.com/article/10.3390/brainsci13020302/s1, Table S1. Grouping of cancer diagnosis by ICD-10 codes. Table S2. Sensitivity analysis of Hazard Ratio of cancer diagnosis by group, subgroup, and type of cancer; Figure S1. Forest plot of Hazard Ratios based on sensitivity analysis.

## Abbreviations

CG	Comparator group
COPD	Chronic obstructive pulmonary disease
DA	Disease Analyzer
GI	Gastro-intestinal
HPA	Hypothalamic–pituitary–adrenal
HR	Hazard ratio
IR	Incidence rate
