# Supplementary material for: Depression Is Associated with an Increased Risk of Subsequent Cancer Diagnosis: A Retrospective Cohort Study with 235,404 Patients
Source: Brain Sci. 2023 Feb 10;13(2):302. doi: 10.3390/brainsci13020302 (PMC9954234; doi:10.3390/brainsci13020302)
Supplement: Supplementary file 1 [file brainsci-13-00302-s001.zip › brainsci-2182792-supplementary.pdf]

Supplementary Material

Table S1: Grouping of cancer diagnosis by ICD-10 codes.

Cancer group	ICD-10 codes
gastro-intestinal	C16-C21
lung	C34
skin	C43-C44
breast	C50
prostate	C61
urinary	C64-68
lymph/blood	C81-96
other*	all else in C

*includes ovarian cancer

Table S2: Sensitivity analysis of Hazard Ratio of cancer diagnosis by group, subgroup and type of cancer.

	Number of events		Comparative risk			
	Patients with depression	Patients without depression	Patients with depression vs. patients without depression (ref)			
Group	N events (%[1])	N events (%[1])	HR	96% CI (lower-upper)		p-value
any cancer diagnosis[2]	3.6%	3.2%	1.14	1.09	1.19	<.0001
by age group (in years)						
18-30	0.4%	0.3%	1.16	0.79	1.69	0.4567
30-40	0.7%	0.5%	1.35	1.03	1.78	0.0311
40-50	1.5%	1.1%	1.33	1.10	1.60	0.0035
50-60	2.9%	2.3%	1.26	1.12	1.41	<.0001
60-70	5.1%	4.2%	1.22	1.12	1.34	<.0001
70-80	7.6%	6.5%	1.18	1.08	1.29	0.0004
80-90	9.2%	8.2%	1.15	1.06	1.26	0.0014
>90	8.1%	7.2%	1.12	0.93	1.36	0.2231
by sex						
female	3.6%	2.9%	1.22	1.15	1.29	<.0001
male	3.6%	3.5%	1.04	0.97	1.11	0.3104

1: percentage of events calculated as number of patients with at least one event divided by all patients within subgroup (by age, sex, type of cancer)

2: any cancer diagnosis includes cancers of the breast, lung, prostate, skin, digestive system including stomach and colon, bladder and kidneys, female reproductive organs including uterus, cervix, ovaries, lymph and blood, and primary cancers of known location as well as malignant secondary of unknown primary origin

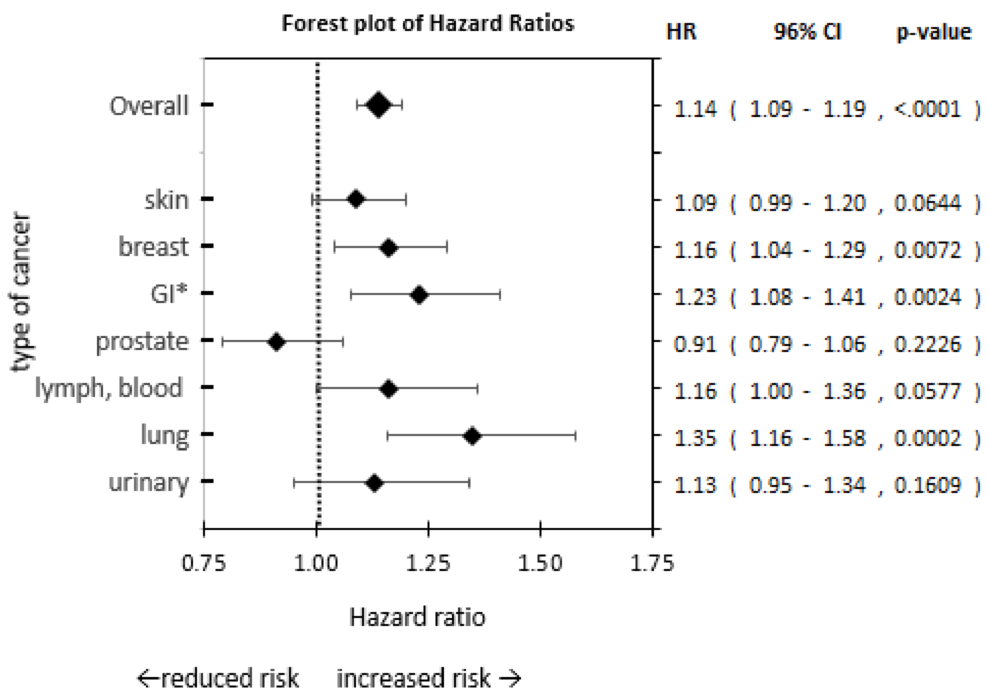

Figure S1: Forest plot of Hazard Ratios based on sensitivity analysis.

Risk of cancer diagnosis was compared between patients with depression and patients without depression (ref) across subgroups by cancer type. Any cancer diagnosis within the first 12 months after index date was censored.

*GI: gastro-intestinal
